# Atomistic insight into the aggregation of [Au_25_(SR)_18_]^*q*^ nanoclusters†

**DOI:** 10.1039/d0na00213e

**Published:** 2020-05-18

**Authors:** Mirko Vanzan, Marta Rosa, Stefano Corni

**Affiliations:** Department of Chemical Sciences, University of Padova Via Marzolo 1 35131 Padova Italy; CNR Institute of Nanoscience Center S3, via G. Campi 213/A Modena 41125 Italy stefano.corni@unipd.it

## Abstract

Atomically precise nanoclusters have been proven to give solid state aggregates with intriguing optical properties. However, the mechanism that regulates this aggregation remains unclear. Here, the aggregation of two Au_25_ nanoclusters in solution is investigated through enhanced sampling molecular dynamics simulations. To understand how the free energy of the systems depends on the nanocluster features, calculations were performed on three nanocluster pairs which differ in charge states and substituent nature and dimension. Our results show that the choice of the ligands heavily affects the free energy profile of the systems when the structures are nearby and, in some cases, the formation of a dimeric phase is observed. This phase is particularly stable in long-chain substituted nanoclusters, where the long alkane chains can generate bundles and the gold cores are closer compared to the short-chain ligands. We found a remarkable agreement between our calculations and the literature-available solid-state structures, especially for the orientation of the interacting nanoclusters. Moreover, some of the dimeric structures are prodromal to the formation of the aurophilic intercluster bond observed in the crystal structures, meaning that the dimer can act as a precursor and can drive the whole crystallization mechanism toward the formation of stable crystal species.

## Abstract 2

Here we investigate the aggregation of Au_25_(SR)_18_ nanoclusters in solution, demonstrating that long-chain alkane substituents can lead to more stable and compact aggregates compared to the case of short-chain ligands.

## Graphical abstract

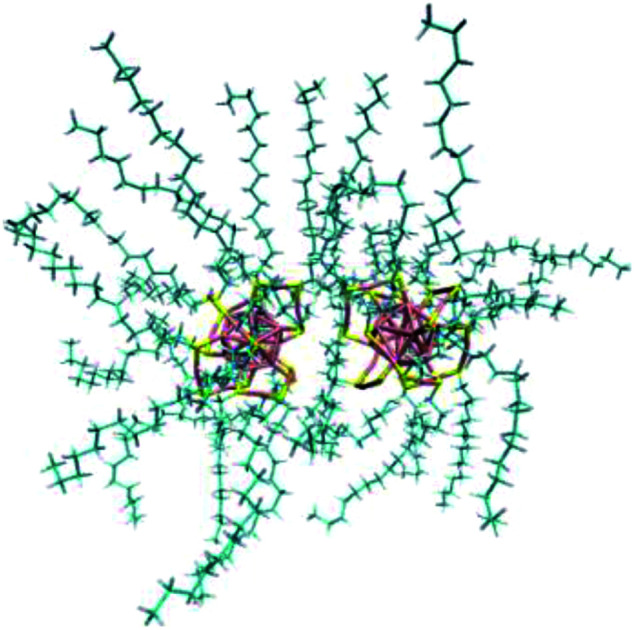

## 1. Introduction

**Fig. 1:**
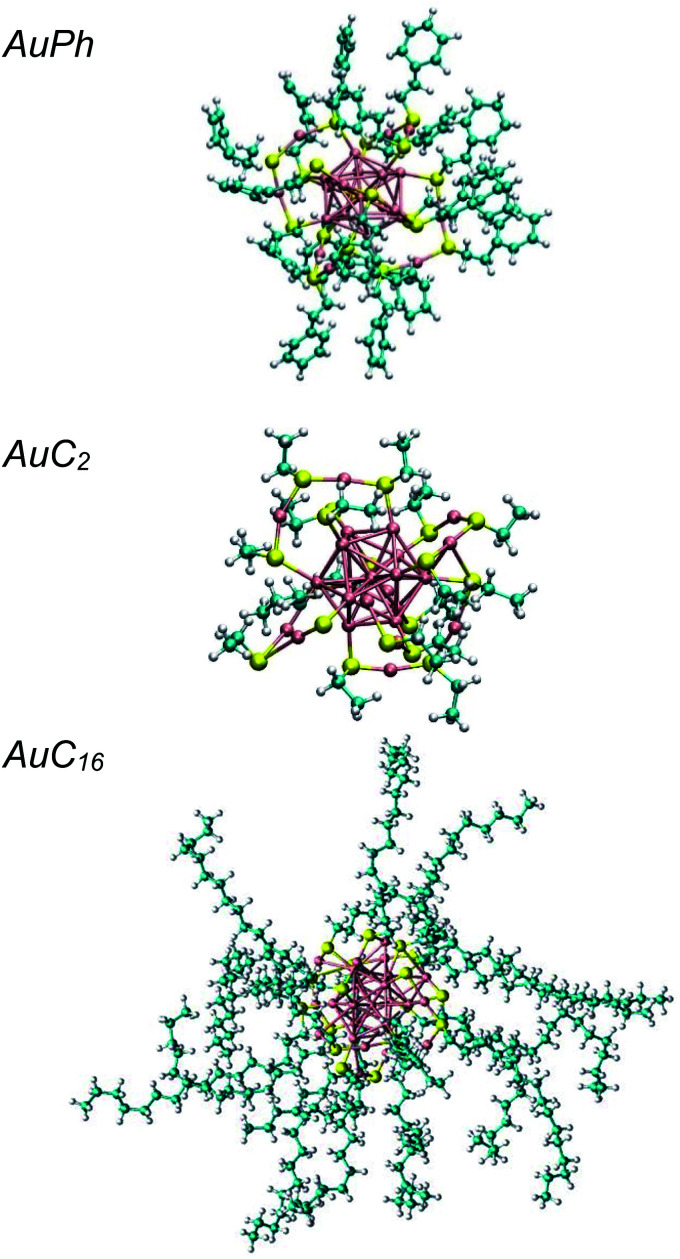
Graphical representations of the investigated nanoclusters. From top to bottom: AuPh = [Au_25_(SCH_2_CH_2_Ph)_18_]^−1^, AuC_2_ = [Au_25_(SCH_2_CH_3_)_18_]^0^, AuC_16_ = [Au_25_(SC_16_H_33_)_18_]^0^. Au atoms are coloured in pink, S in yellow, C in azure and H in white.

The study of the chemical and physical properties of monolayer-protected gold nanoclusters (MPCs) has recently attracted much attention in the field of nanoscience, as they exhibit unusual physical and chemical properties which are directly related to their peculiar geometries.^1^ In these nanostructures, the inner interatomic interactions compel the gold atoms to occupy a small volume, giving rise to strong quantum confinement effects and, as a consequence, to unique optical and magnetic properties which can be further tuned by modifying the dimension of the metallic part and the nature of the organic ligands.^2–9^ This makes them promising candidates for many practical applications, such as catalysis, energy conversion, nanosensors and nanomedicine.^10–13^ Among all stable MPCs, [Au_25_(SR)_18_]^*q*^ (R = organic ligand) received the most extensive attention both in experimental and computational studies, as they can be efficiently synthesized through simple chemical reactions^14^ and the relative X-ray resolved crystal structures are known for more than 10 years.^15^ The computational approach generally agrees with the experimental results on MPC diluted solutions (experimental studies are usually performed in the μM or mM range^14^), however it fails to predict the optical features of systems where the local concentration of the nanoclusters is high enough to generate solid aggregates. Recently, several groups have highlighted that under certain conditions, *e.g.* in phospholipid membranes,^16^ human monocytic cells^17^ or amorphous films,^18^ MPCs can generate aggregates whose optical properties are remarkably different from those of the isolated nanoclusters (*e.g.* increased two photon absorption cross-section). Some insight on the aggregation process was recently obtained through coarse-grain calculations of the nanoclusters in water,^19–21^ while a relatively small number of studies focused on investigating the properties of atomically precise nanoclusters at the atomistic level and, in any case, they never account for the dynamics of multi-cluster systems.^22–25^ Moreover, the effect of apolar solvents on cluster aggregation was never investigated, despite being the ones commonly used in their preparation. In this study we explore the aggregation of these nanoclusters at the atomistic level, by investigating the formation of cluster dimers, which are the minimal aggregate units. We chose to perform molecular dynamics simulations on three different systems composed of two identical MPCs in dichloromethane, which is a largely employed solvent for MPCs.^26–30^ The studied structures, which are represented in Fig. 1, are:

• AuPh: mono-negative charged clusters [Au_25_(SCH_2_CH_2_Ph)_18_]^−1^ (Ph = phenyl)

• AuC_2_: short linear carbon chain substituted neutral clusters [Au_25_(SCH_2_CH_3_)_18_]^0^

• AuC_16_: long linear carbon chain substituted neutral clusters [Au_25_(SC_16_H_33_)_18_]^0^

These three systems were chosen in order to test how different charge states and substituent nature and length can affect the equilibrium features of the solution and therefore their aggregation properties. It is indeed well known that the substituent structure can affect the stability of the nanoclusters which, under some conditions can modify their inner structure upon ligand exchange.^31^ However, their impact on the aggregation features is still unclear. Moreover, AuPh and AuC_2_ systems are well known in the scientific community for the past several years and can be used to perform accurate comparisons between our results and the experimental data.^16,28,32–38^ We characterized the aggregation features of these MPCs in solution using metadynamics simulations, enhancing the sampling along the cluster–cluster interdistance that we chose as a collective variable of our simulations.^39^ This method allowed us to explore the whole phase-space configurations of the mutual nanocluster distance and to make predictions on the stability of the aggregates. Through this approach, we were able to characterize the equilibrium properties of the three solutions and to give new and innovative points of view on the cluster aggregation dynamics.

## 2. Results and discussion

### 2.1 Analysis of the free energy landscapes

**Fig. 2:**
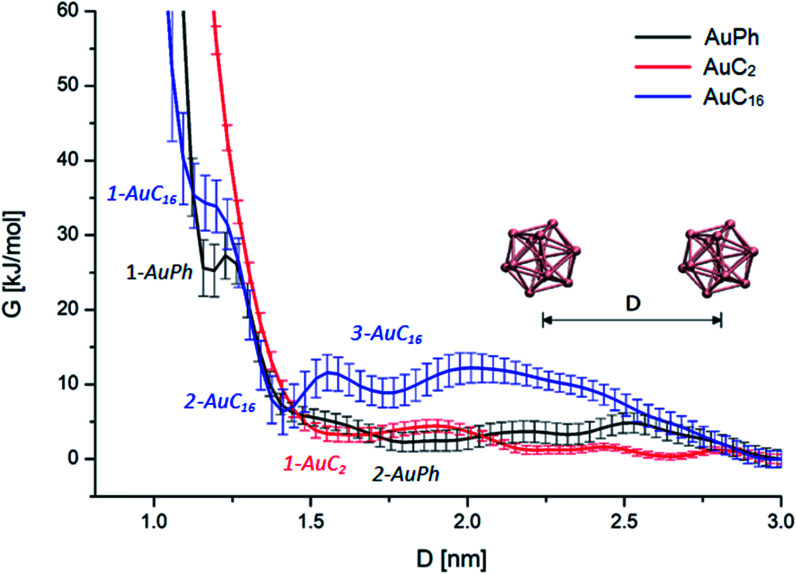
Free energy profiles of the three analysed MPC systems with respect to the mutual cluster–cluster distance. Black, red and blue curves represent the FES of AuPh, AuC_2_ and AuC_16_ respectively. Curves are aligned in order to set *G* = 0 at *D* = 3 nm.

First of all, we wanted to inspect the free energy profiles of the three studied systems in order to identify the most stable configurations and the differences in the overall behaviour of the nanocluster dynamics. To ease the comparison, the free energy profiles were aligned to zero at *D* = 3 nm (at this distance the MPCs can be considered isolated). In the following, free energy values Δ*G* will always be reported with respect to this zero-point energy. From the free energy surfaces (FES) reported in Fig. 2, we can understand many features of the thermodynamic stability and the physical arrangement of the nanoclusters. As a first consideration, all systems have their global minimum with the two MPCs in a non-interacting configuration (*D* ≥ 3 nm). However, there are shallow but noticeable local minima for the interacting configurations, whose positions and depths are summarized in Table 1. Although the obtained Δ*G* values are positive, some of them are very small, amounting to a few *k*_b_*T*. This means there will be an equilibrium between the dimers and the free clusters in solution at room temperature, with both forms present. Considering that the clusters are soluble in the solvent we are considering (*i.e.*, the free form should be predominant) this is a sound result. More importantly, we found that there is a free energy barrier (although small) to dissociate the dimers, which means that once the dimers are formed they can remain in this configuration for some time in solution. Looking at Fig. 2, we can infer the following features:

**Table 1:** Quantitative characterization of FES minima. Δ*G* is the value of free energy at the minimum, calculated with respect to the free energy at *D* = 3 nm

Minimum	AuPh		AuC_2_	AuC_16_		
	1-AuPh	2-AuPh	1-AuC_2_	1-AuC_16_	2-AuC_16_	3-AuC_16_
*D* [nm]	1.20	1.80	1.66	1.18	1.41	1.73
Δ*G* [kJ mol^−1^]	25 ± 3	2 ± 1	3 ± 1	34 ± 3	6 ± 3	9 ± 2

AuPh: the profile shows a small minimum at *D* = 1.20 nm (named 1-AuPh in Fig. 2). This intercluster distance corresponds to a dimeric configuration stabilized by π–π stacking interactions occurring between the phenyl rings of the two nanoclusters, as visible in Fig. S1.† Minimum 1-AuPh is anyway difficult to access, as the system needs to overcome a large free energy barrier, making the non-interacting configuration the most favourable. The presence of this minimum is nevertheless interesting, first for the sake of comparison with the other systems and then because it highlights the importance of the interaction between ligands in determining metastable structures, even where the coulombic repulsion is at work (the clusters are charged). Apart from this configuration, there is another slightly noticeable minimum at *D* = 1.80 nm (2-AuPh in Fig. 2), which may correspond to the distance where the coulombic repulsion and the attractive non-bonding interactions acting between the two nanoclusters have similar magnitude. Above this region, the profile can be considered substantially flat, meaning that the structures are free to move in a diffusive way.

**Fig. 3:**
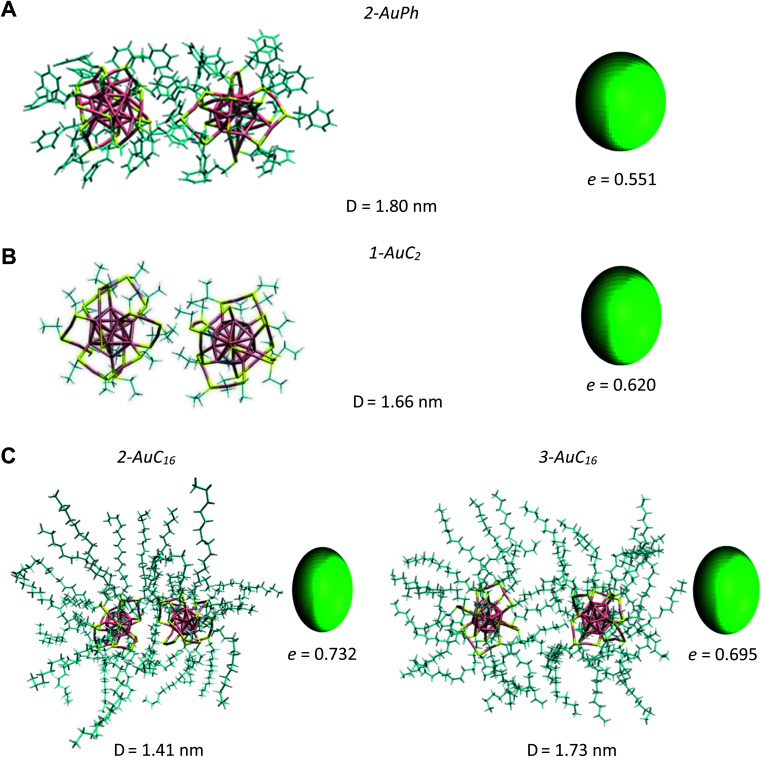
Snapshots of the analysed systems in their close-cluster minima. From top to bottom: (A) AuPh (2-AuPh), (B) AuC_2_ (1-AuC_2_), (C) AuC_16_ (2-AuC_16_ and 3-AuC_16_). Solvent molecules are omitted for clarity. Each panel contains an ellipsoid model of the nanoclusters, whose eccentricity *e* was calculated as the average of the instantaneous clusters' eccentricities at that distance.

AuC_2_: the FES shows a shallow minimum (1-AuC_2_ in Fig. 2) at *D* = 1.66 nm. This is probably related to the non-bonding interactions between the two nanostructures. The main difference from the other two systems is that here we do not observe any minimum at shorter distances, like minima 1-AuPh and 1-AuC_16_. This can be explained on the basis of the nature of the organic substituents. Indeed, unlike other substituents, ethyl chains are too short to generate stable dimeric configurations and bounded frameworks (see Fig. 3, panel B). Similar to what was said for minimum 2-AuPh, with such a shallow minimum the free energy profile can be considered almost flat with the nanoclusters free to move in a diffusive way.

AuC_16_: in this case, the free energy landscape shows a more complex structure. Indeed, looking at the profile in Fig. 2, there are three notable points: a barely visible plateau at 1.18 nm (1-AuC_16_), a pronounced minimum at 1.41 nm (2-AuC_16_) and another smoother minimum at 1.73 nm (3-AuC_16_). These points represent configurations in which the interligand interactions between the long chains stabilize the dimeric phase. Configuration 1-AuC_16_ corresponds to a region where the long chain interdigitation is strong enough to compete with the short-range repulsive interactions. As discussed for minimum 1-AuPh, this configuration is very difficult to reach because of its high free energy (Δ*G* = 34 kJ mol^−1^), but its existence highlights the importance of the ligand interaction in generating metastable aggregates. Regarding minima 2-AuC_16_ and 3-AuC_16_, it can be seen that in both of them the organic chains organize creating bundles which stabilize the dimeric phase, as confirmed by the visual inspection reported in panel C of Fig. 3. Even if these minima do not represent the most stable state, which is instead the non-interacting configuration (*D* > 3 nm), the barriers which separate the three minima are approximately 5 times *k*_b_*T*. Thus, they are small enough to be possibly overcome at room temperature, allowing the system to assume close-cluster configurations and generate relatively stable dimers. The position and the depth of these minima provide an atomistic rationale to the experimental evidence of stable aggregate formation when long-chain substituted Au_25_ are dispersed in a polymeric film.^19^

It is interesting to note how the length of the substituent affects not only the depth, but also the position of the interacting configurations: from our study we can see that systems with longer substituents give more stable dimeric configurations. Indeed, the minima features are mainly related to a direct interaction between the organic chains of the two nanoclusters, which dock the two clusters into a dimer. This is evident if we consider the presence of configurations 1-AuPh and 1-AuC_16_.

They can be explained only by taking into account the effect of the interligand interaction, whose magnitude is high enough to modify the FES at distances where the repulsion among nanoclusters is strong (particularly in the system AuPh where repulsive coulombic interactions are present, see the ESI† for details on its magnitude). Thus, we can state that the π–π stacking interactions observable in the system AuPh and the van der Waals forces acting between the long alkyl chains in the system AuC_16_ contribute in a similar way to the stabilization of the system at very short distances. All these considerations have to be rationalized taking into account the effect of the environment. Indeed, if the two nanoclusters are interacting effectively one would expect a free energy minimum located in the region where the clusters are interacting. However, here the solvent plays a non-negligible role in determining the stability of the system. In particular, we have to account that since the nanoclusters are soluble in the considered solvent, the nanoclusters–solvent interaction tends to stabilize the isolated cluster configuration. When the nanoclusters become closer, the whole Solvent Accessible Surface Area (SASA) of the system decreases and thus part of these favourable interaction is lost. To quantify this effect, we have evaluated SASA as a function of the intercluster distance *D* for the system AuPh, and we found that the loss of SASA for each AuPh nanocluster at *D* = 1.20 nm is about 20% of the SASA of isolated clusters. More details are available in the ESI.†

As the stability of the interacting configurations and the capabilities of the nanocluster to aggregate may depend on the adopted force field, we compared the results obtained on the system AuC_2_ with those obtained with an AMBER-based force field.^22^ As visible from the free energy landscape reported in Fig. S2,† this force field allows for the generation of a short-range minimum (*D* = 1.45 nm) which is not present in the AuC_2_ profile represented in Fig. 2. However, this new profile suffers from important computational artefacts (we observe that the nanoclusters started to break into smaller fragments during the simulation) which preclude a meaningful analysis. A detailed discussion of the results obtained using this force field is available in the ESI.†

### 2.2 Comparison with the crystal structures

**Fig. 4:**
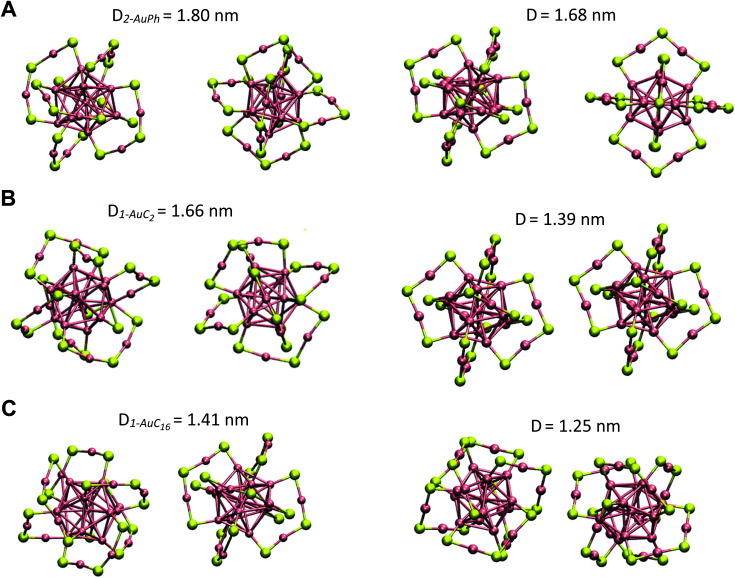
Comparison between the Au_25_S_18_ backbone of the three nanocluster systems in their dimeric configurations (left side) and their relative X-ray resolved crystal structures (right side) taken from reference.^33,40^ From top to bottom: (A) AuPh (minimum 2-AuPh), (B) AuC_2_ (minimum 1-AuC_2_), (C) AuC_16_ (minimum 2-AuC_16_). (D) Distance between the centres of the two Au_13_ kernels.

Since we are not aware of experimental insights on gold nanocluster configurations in solution, we compare our data with solid-state crystal structures. In Fig. 4 we report the Au_25_S_18_ backbone of our three nanocluster systems in their dimeric configurations recovered from trajectory snapshots, and the ones recovered from the available crystal data. Among the different minima of the three systems, we chose for each of them the one which could be more reasonably compared with the experimental data, *i.e.* the interacting configurations with the lowest associated Δ*G* value. This means minima 2-AuPh (*D* = 1.80 nm), 1-AuC_2_ (*D* = 1.66 nm) and 2-AuC_16_ (*D* = 1.41 nm) for AuPh, AuC_2_ and AuC_16_ systems, respectively. Concerning the choice of experimental systems, we compare AuPh ([Au_25_(SCH_2_CH_2_Ph)_18_]^−1^) with the crystal structure of [Au_25_(SCH_2_CH_2_Ph)_18_]^0^ taken from reference.^40^

Despite being negatively charged, this structure does not suffer from the presence of bulky counter ions which are naturally present in crystals of charged MPCs, whose presence modifies the distance between the nanoclusters and their orientation in the solid phase. AuC_2_ results were directly compared with its crystal structure,^33^ while data coming from AuC_16_ ([Au_25_(SC_16_H_33_)_18_]^0^) were compared with the crystal structure of linear carbon chain substituted [Au_25_(SC_5_H_11_)_18_]^0^, taken from reference.^40^

Starting from the distance between the clusters, which is reported in Fig. 4, it is visible how our calculations provide larger values than in the crystal. In particular, the calculated inter-cluster distances are 0.12 nm and 0.16 nm longer for systems AuPh and AuC_16_ respectively, while this difference reaches 0.27 nm in the case of the ethyl substituted nanoclusters. These overestimations can be justified by taking into account various effects. First of all, it has to be noted that our data refer to solutions which are naturally affected by a higher entropy compared to the solid state. This extra entropy affects the thermodynamics of the systems by modifying the equilibrium distances and increasing the width of the distribution of the mutual distance between the clusters in the dimer. Second, AuPh and AuC_16_ systems are compared with crystals of MPCs with different charge states and substituent lengths and these certainly affect the cluster–cluster distances in the crystal structure. Moreover, we are comparing stable solid-state crystal structures with metastable dimeric phases which are significantly different. For example, the dimer associated with minimum 2-AuPh is unstable since even thermal energy can break this phase (Δ*G* = +2 kJ mol^−1^) and bring the system to its global free energy minimum (isolated cluster configuration), meaning that its formation is transient and thus cannot be fully compatible with a stable crystal structure. Last but not least, it has to be emphasized that all interactions were represented with a classical force field, which, by its own nature, cannot capture subtle effects related to the quantum mechanical nature of the systems, *e.g.* the generation of intercluster aurophilic bonds. Despite all the aforementioned aspects, there are several similarities among the calculated and the experimental structures. First of all we can notice how calculations perfectly reflect the distance trend noticed in the crystal phase: long-chain substituted nanoclusters have shorter cluster–cluster distance compared to the ethyl substituted system, which in turn has a shorter distance than the phenyl substituted ones.

From Fig. 4 it is also evident how in the case of AuPh and AuC_16_ the dimeric phases and the solid-state structures show very similar relative orientations, while for AuC_2_ the two structures differ on the spatial arrangement of the Au_25_S_18_ backbone. Regarding AuC_2_, the crystal structure shows a centrosymmetric cell containing only one nanocluster,^33^ meaning that no real dimer configurations are present in the crystal phase. This fully agrees with our previous considerations about AuC_2_ dimer stability and justifies the difference in the geometry reported, as neither the experiment nor the simulation presents a stable interacting geometry that can allow for meaningful comparisons. We could think that the same argument could be applied to AuPh, as the computed 2-AuPh minimum is very similar to 1-AuC_2_. Nevertheless, here the comparison between simulation and experiments is less straightforward due to the different charges of the two systems, which could favour an interacting configuration in the experiment. We were able to find geometries belonging to 2-AuPh minimum which are oriented similar to the experimental result, showing that this configuration can be present both in the neutral and in the negatively charged systems. We can only speculate that with neutral nanoclusters this orientation would become dominant, like what we observed for the AuC_16_ system, where the similarity between computational and experimental data is remarkable. For the specific case of system AuC_16_, we were able to perform a meaningful numerical analysis to compare the relative crystal structure orientation with the computational results. This was possible neither for AuPh, where the different charges of the simulated system and the experiment prevent a consistent comparison, nor for AuC_2_, where the clusters do not generate a proper dimeric structure, as already discussed. Therefore, focusing on the system AuC_16_ and in particular on the free energy minimum 2-AuC_16_, we sampled the dihedral angle defined by S^α^–Au^α^–Au^β^–S^β^ (where α and β refer to different nanoclusters) which is the dihedral angle between the staples of the two clusters, describing their relative orientation. This angle, calculated from the trajectory, was found to be 102 ± 16°. Taking into account the different stabilities of the dimeric structure due to the different environments, this value is nicely comparable with that of the crystal structure reported in Fig. 4, which is *ca.* 90°.

### 2.3 Insight on the aggregation

To further investigate the interacting configurations of the system AuC_16_, we can exploit the fact that atomistic simulations allow us to study not only the structures of the aggregates' configurations but also the mechanism that leads to their formation. If we perform a visual inspection of the trajectory frames where the two long-chain MPCs are in their interacting region (*D* < 2 nm), we notice that, to reach the dimeric configuration, one nanocluster has to move towards the other by modifying is orientation and by moving the organic substituents in order to minimize the steric hindrance among the nanocluster staples. Therefore, the ability of the nanoclusters to interact effectively and to give rise to quite defined dimeric structures is controlled by the orientation of the two nanostructures which move and reorganize their structures before approaching each other. We notice that there are some specific relative orientations that allow the cluster–cluster locking mechanism. The formation of the dimer is thus a two-step process, *i.e.*, a “twist and lock mechanism”.^24^ In the first step (twist) there is a reorientation of the nanoclusters, which is mandatory for an effective core–core interaction. In the second step (lock) the clusters become closer and reach the dimer configurations corresponding to the relative free energy minimum. Our result represents an atomistic evidence for this “twist and lock” mechanism, which was proposed in the previous study.^24^ In this mechanism, when the golden cores start to move towards each other, there is a reorientation of the whole structure aimed to minimize the distance between two gold staple atoms and that allows for the interdigitation of ligands. This reorientation brings the systems in a dimeric geometry where the two clusters are locked in a single structure. As far as we know, this non-trivial result represents the first atomistic proof of the mechanism behind the formation of aggregates and suggests that the orientational configuration can play a primary role in determining the aggregates' stability and, in turn, the physical properties, *e.g.* by allowing new optically active transitions.

Despite the differences in the mutual distances, our result demonstrated that regardless of the nature of the MPCs, the interaction configurations in solutions are similar to the ones in the crystal structure, and the dimer formation could establish a reliable starting point to understand the nucleation mechanism behind the crystallization process. Please note that the presence of metadynamics bias does not affect the nanocluster reorientation motion since it acts as a driving force only on the mutual intercluster distance and thus cannot influence in any way the orientational configuration. However, here we observe that the rearrangement of alkyl chains during the twist and lock mechanism is relatively sluggish for the longer chains (AuC_16_). Thus, in this particular case sampling would benefit by enhancing such orientational motions. To this aim, combining replica exchange with metadynamics would be a reasonable (although computationally demanding) option.

The aforementioned evidence on the orientational motions stimulated further insights on the possibility of nanocluster crystallization and its dependency on the chain lengths. To better understand the behaviour of the different systems, we selected from our simulations on systems AuC_2_ and AuC_16_ geometries belonging to their most stable dimeric configurations. In particular we investigated the dimeric structures of minima 1-AuC_2_ and 2-AuC_16_, by including all configurations within a distance interval of ± 0.1 nm with respect to the free energy minima. For all these geometries we calculated the minimum distance between gold atoms belonging to the two MPCs in solution. These distances were found to be 7.2 ± 0.9 Å for AuC_2_ and 4.4 ± 0.6 Å for AuC_16_. These values confirm how long-ligand systems are characterized by closer intercluster distances with respect to the ethyl substituted case, where an appropriate dimeric interaction configuration is absent. Furthermore, the presence of a mildly stabilized phase in AuC_16_ when *D* = 1.18 nm (configuration 1-AuC_16_) indicates that this system is more prone to give configurations where the MPCs are even closer and where the nanoclusters can have the time to self-arrange, allowing the formation of intercluster Au–Au bonds. Moreover, in the configuration 1-AuC_16_ the minimum distance between gold atoms belonging to the two clusters is about 3.6 ± 0.5 Å which is indeed coherent with the distance observed in the crystal structure [Au_25_(SC_5_H_11_)_18_]^0^ which is about 3 Å.^40^ These results are coherent with the tendencies observed in the experiments. Indeed while long-chain structures can generate wires where two nanoclusters are connected through an aurophilic bond,^40^ short-chain clusters do not have this capability.^33^ Of course, our calculations cannot capture the formation of aurophilic bonds, as already discussed. However, with these results we can confirm the counterintuitive fact that long substituents lead to short average intercluster Au–Au distance and especially, to dimeric geometries which are coherent with the solid state structures. Indeed, the different standard deviations associated with the intercluster distance reflect the larger variety of relative conformation assumed by the short chain substituted nanoclusters (which are almost free to move in a diffusive way), compared to the relatively stable configuration obtained in the other case. Finally, experimental evidence demonstrated that [Au_25_(SC_16_H_33_)_18_]^0^ nanoclusters are poorly soluble in dichloromethane and tend to form aggregates.^41^ This observation is fully compatible with the existence of minima 2-AuC_16_ and 3-AuC_16_ in Fig. 2. Indeed, the calculated free energy landscape shows that the dimeric configurations associated with these minima are relatively stable and easily obtainable at room temperature, meaning that the aggregation in this system cannot be neglected.

### 2.4 Modelling MPC shapes: the ellipsoid representation

A visual inspection of the trajectory snapshots reveals that the different dynamics of neutral nanoclusters rely on the flexibility of the long ligand chains of the system AuC_16_ with respect to the ones of the system AuC_2_. Indeed, a linear alkane C_16_ chain is quite flexible and can self-arrange forming bundles, allowing a unique and shorter intercluster Au–Au distance (see Fig. 3, panel C). This is not possible with the ethyl substituents which are more rigid and have less orientational degrees of freedom. A possible method to quantify the geometrical rearrangement of the structures in their dimeric configurations is by computing the dependency of nanocluster moment of inertia^42^ with respect to the mutual cluster distance. By recovering the eigenvalues of the moments of inertia tensor it is possible to obtain information about the shape of the whole nanostructure for each trajectory frame. It is important to consider the whole nanoclusters since ligands can have a major role in determining the shapes of structures. For example, the diagonalized moment of inertia matrix of a nanocluster where ligands are highly elongated (prolate ellipsoid) has one major component along the elongation direction and two minor components along the orthogonal directions. In order to make comparisons among the three systems, it is mandatory to have a geometrical parameter which does not depend on the mass of the nanostructures. Therefore, we decided to model the clusters as moving ellipsoids and calculate their eccentricity as a function of the mutual cluster–cluster distance as reported in the following equation:1
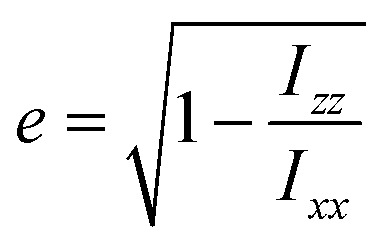


**Fig. 5:**
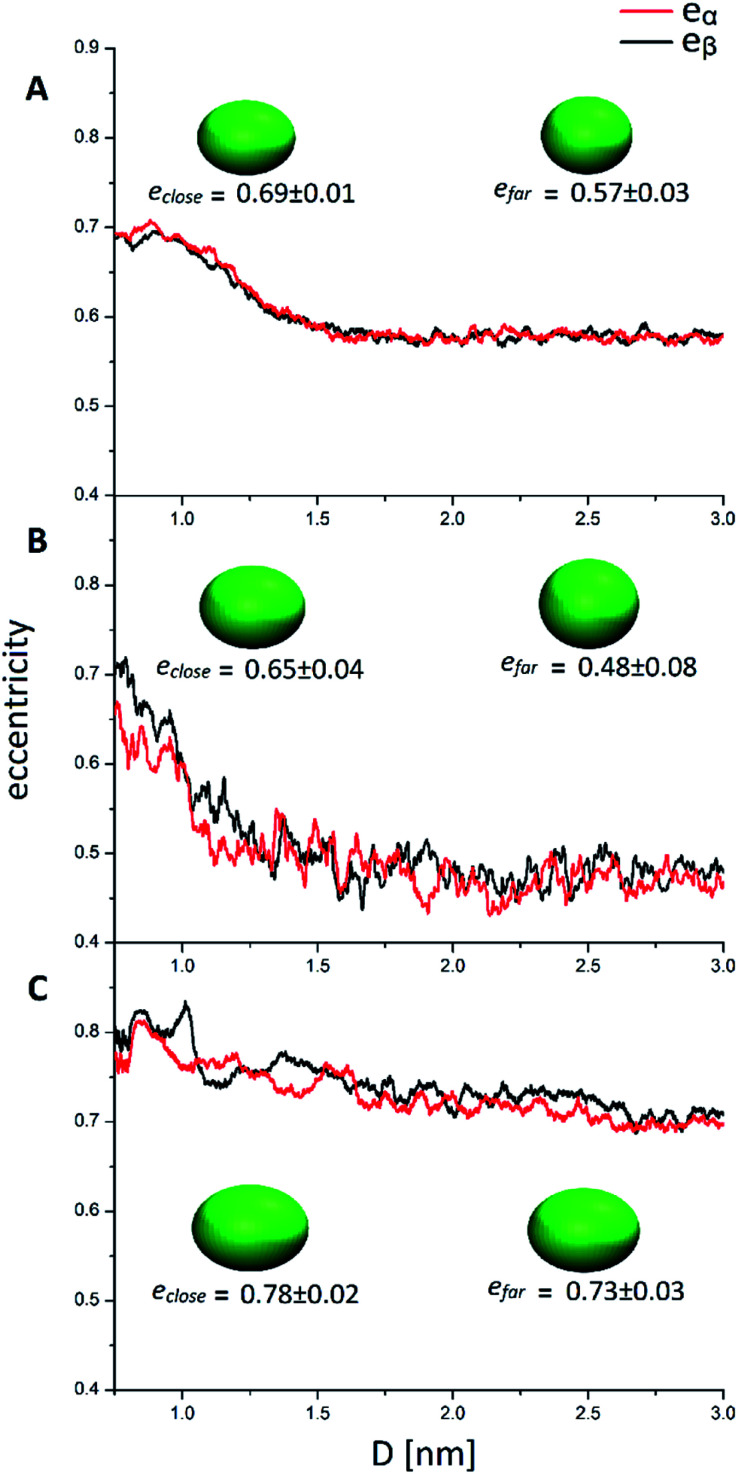
Eccentricity of the two nanoclusters (α and β) in solution with respect to the mutual cluster distance. (A, B and C) refer to AuPh, AuC_2_ and AuC_16_, respectively. The inset ellipsoids represent the average shape of the two MPCs in close (*D* < 1 nm) and far (*D* > 2 nm) regions.

The quantities *I_xx_*, *I_zz_* are the eigenvalues of the moment of inertia tensor calculated along the *x* and *z* axes respectively, and *I_xx_* > *I_yy_* > *I_zz_.* The rationale was to choose the two directions of maximal and minimal sizes, so as to provide an estimate of the maximal deviation of the cluster shape from a sphere. The parameter *e* contains important information on the average shape of the clusters along the trajectories. In particular, the higher its value, the more prolate is the ellipsoid. In contrast, smaller the value, more spherical-like is the geometry. Because of its definition, expressed in eqn (1), the value of *e* has a non-linear dependency on the semi-axis length, since they are proportional to the square root of the moment of inertia. This reflects in a non-linear connection between *e* and the ellipsoid shapes. For example, if we consider ellipsoids with *e* < 0.5, it is very difficult to notice the difference from a sphere by visual inspection. In contrast, ellipsoids with high values of *e* are highly prolate, and higher the value of *e* more pronounced is the elongation. For this reason, we will consider *e* = 0.5 as the threshold to determine if a nanocluster has a spherical or elongate geometry. Please note that *e* = 0.5 means that one major semi-axis is longer than the other by about 15%. The way the eccentricity depends on the inter-cluster distance is reported in Fig. 5. To facilitate the discussion, we report more significant values of these quantities in Table 2.

**Table 2:** Maximum (*e*_max_), minimum (*e*_min_) and averaged values of the eccentricity of the clusters in their close (*e*_close_) and far (*e*_far_) cluster configurations. *e*_max_ were always recovered when 0.8 < *D* < 0.9 nm. *e*_min_ were always recovered when *D* > 2.3 nm. Error bars represent the standard deviation of the values

	AuPh	AuC_2_	AuC_16_
*e*_max_	0.81	0.79	0.93
*e*_min_	0.26	0.24	0.36
*e*_close_ (0.8 nm < *D* < 1 nm)	0.69 ± 0.01	0.65 ± 0.04	0.78 ± 0.02
*e*_far_ (2 nm < *D* < 3 nm)	0.57 ± 0.03	0.48 ± 0.08	0.73 ± 0.03

Looking at Fig. 5, it can be easily understood that in all systems, higher eccentricity is obtained when the nanoclusters are close to each other (*D* < 1 nm). However, the way they reach the maximum eccentricity is different and it is directly related to the interactions among the nanoclusters. In the system AuPh, the increase of *e* is related to the chain reorganization arising from the π–π driving force, which stabilizes the close-cluster structures where the phenyl rings are paired, giving the nanoclusters a more prolate configuration, as visible in the inset of Fig. 5 panel A. Regarding the system AuC_2_, the high eccentricity variation can be related to the geometry of the free structure itself. Indeed, when the structures are isolated (*D* > 2 nm) their eccentricities are very small (even below 0.50) and their shape can be approximated to a sphere. When moving towards the other nanoclusters, the ligands start to move in order to facilitate the approach and the shape changes drastically, giving rise to a more elongated ellipsoid as visible in the inset of Fig. 5 panel B. Note that while in AuPh the two present a practically symmetrical decrease in the eccentricities moving from a close-cluster configuration to an isolated cluster regime, this is not completely true for the other two systems where the MPCs seem to have slightly different behaviour. This can be explained by considering the motion and nature of the substituent chains. Due to the π–π stacking interaction, the phenyl rings tend to move in a less chaotic way than the alkyl chains. As a consequence, in AuC_2_ and AuC_16_ the eccentricity curves present more fluctuation compared to the AuPh system and this reflects a non-symmetrical trend of the eccentricity.

Focusing now on the system AuC_16_, in Fig. 5 panel C it is visible how the average eccentricity is higher than in the previous cases, both in the close-cluster and the far-cluster regime (see Table 2). This means that these nanoclusters are naturally prone to give elongated shapes, even if they are isolated. Such behaviour can be explained on the basis of van der Waals interaction acting between the ligands, which tend to elongate the geometries along a preferential direction. These van der Waals interactions together with the length of the chains themselves, could also explain why in Fig. 5 panel C the eccentricities present a very smooth decrease with respect to the inter-cluster distance. Indeed, the messy movement of the chains together with their tendency to form bundles gives a softer decrease of the eccentricities of the MPCs. This observation agrees with previous studies made by Antonello *et al.* who demonstrated that, in an apolar solvent, MPCs substituted with alkane linear chains with more than 12 carbon atoms allow the formation of elongated structures.^42^ Moreover, the oscillation visible in Fig. 5 panel C indicates that the long-chain MPCs do not follow a single well-defined pathway to move the substituents and allow the interlocking of the inner atoms, but instead they arrange the substituents in disordered but very elongated geometries because of the presence of isotropic non-bonding interactions among the chains, giving rise to a relatively high uncertainty on the eccentricity.

Finally, starting from the calculated far-cluster eccentricities we estimated the nanoclusters' diffusion coefficients for systems AuC_2_ and AuC_16_ using the relationships described in reference.^43,44^ The estimated diffusion coefficients are *D*_AuC_2__ = 11.2 × 10^−6^ cm^2^ s^−1^ and *D*_AuC_16__ = 4.8 × 10^−6^ cm^2^ s^−1^ while the available experimental data for these nanoclusters in dichloromethane are 
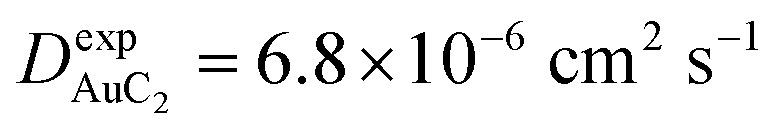
 and 
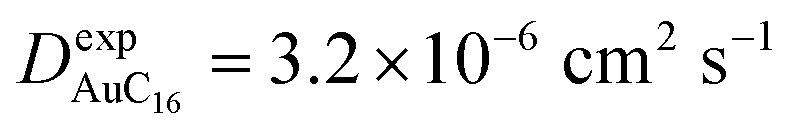
.^41^ Even if the calculated values are both comparable to the ones reported in the literature, it can be noticed that they are slightly overestimated. However, considering the ratio among the diffusion coefficients we obtain 
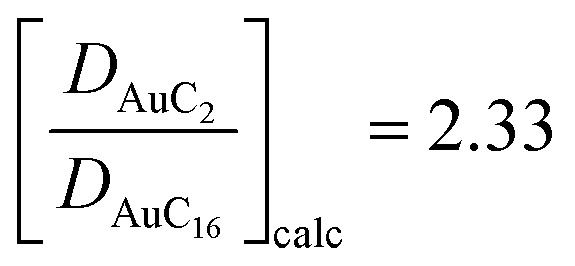
 for the calculations and 
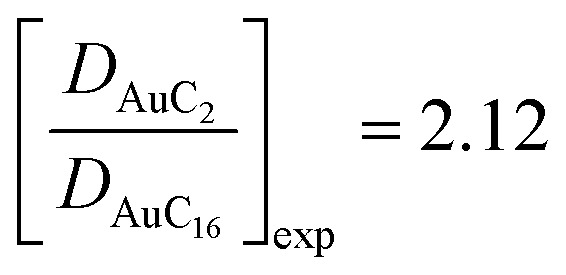
 for the experimental data. Taking into account the uncertainty associated with the nanocluster shapes along the trajectories, the similarity between these ratios is remarkable. This confirms the quality of our calculations in predicting the dynamical properties of these systems and indicates that changing the length of ligand chains from C_2_ to C_16_ can affect their diffusion properties and reduce their mobility by a factor of two.

## 3. Conclusions

In this work we performed molecular dynamics simulations to investigate the aggregation of three different Au_25_-based nanocluster pairs in dichloromethane. In order to quantitatively describe the thermodynamic features of the systems, we calculated the free energy profiles through metadynamics simulations, using the mutual cluster distance as a collective variable. Although the chosen nanostructures differ both in the charge state and the ligand type, our results clearly demonstrate that the most stable configuration is independent of these factors and is obtained when the nanostructures are isolated (*D* ≥ 3 nm). However, the choice of the nature of the ligands and their length heavily affect the thermodynamic features when the structures are close enough to interact (*D* < 2 nm). In particular, our analysis shows that interactions among ligands, such as π–π stacking (system AuPh) or van der Waals interactions (system AuC_16_), can generate mildly stable configurations when the clusters are very close (*D* ≈ 1.20 nm). The aggregation at such small distances still remains highly improbable, but the presence of plateaus in the free energy landscapes evidences the importance of ligand interactions in the whole system dynamics. We then discussed the aggregation properties of neutral nanoclusters, which were found to be strongly dependent on the substituent length. Short-chain substituted structures (AuC_2_) show an almost flat free energy profile even at short cluster–cluster distances (1.4 < *D* < 2 nm), meaning that the MPC approaches in a diffusive way and even the formation of transient aggregates is unlikely. In contrast, long chain neutral clusters (AuC_16_) present two main free energy minima at 1.41 nm and 1.73 nm, both easily accessible at room temperature. The different behavior of the two neutral systems resides in the flexibility of the long chains which can move in order to generate bundles, giving the dimer a highly elongated shape. This is supported by the presence of a shorter and relatively well-defined intercluster Au–Au distance in the AuC_16_ dimers, in contrast to AuC_2_ as also confirmed by available experimental evidence.^40^ A systematic analysis of the dimeric configurations obtained from the simulations reveals an excellent agreement with the available X-ray resolved crystal structures, both in the mutual cluster interdistance and in the orientation of the structures, suggesting that the formation of dimers in solution can represent the first step of the MPC crystallization process. Furthermore, the way the nanoclusters approach each other to build a single dimer is compatible with the “twist and lock” mechanism which was observed here for the first time using an atomistic approach. Finally, by analysing the nanoclusters as moving ellipsoids, we demonstrated that the geometries strongly depend on the mutual distance: the more closer the clusters are, the more they are elongated. This shape elongation is particularly evident in the long chain substituted case, indicating the formation of bundles that stabilise the dimeric phases. The shape of these nanoclusters was quite elongated even in the regions where the clusters are isolated (*D* ≥ 3 nm). This reflects in a slower mobility of these compared to the short-chain case, as confirmed by the estimated diffusion coefficients. Generally speaking, we found that the organic substituents can play a primary role in the aggregation process since long ligand chains can generate compact and stable dimers, making the aggregation more probable compared to the other cases. Although more research has to be made to fully understand the dynamics of these systems, changing for example the solvents or the substituent structure, this study represents a first, solid guideline for further theoretical and experimental investigation on MPC aggregates.

## 4. Computational details

The single nanocluster geometries were obtained from the B3LYP//6-31G/LANL2DZ optimized structure of [Au_25_(SCH_3_)_18_]^0^ presented in reference.^45^ The extra atoms of the substituents were added manually adopting common C–C and C–H bond lengths of aromatic rings and alkanes. All simulations were performed with Gromacs^46^ 5.0.7, patched with PLUMED^47^ 2.4.0 for the metadynamics calculations. Single atom partial charges were attributed following the RESP procedure^48^ on the *ab initio* optimized geometries, as implemented in the code PyRED.^49,50^ Intramolecular bonds and dihedral angles were parametrized as harmonic oscillators while intermolecular forces were modelled on the basis of Lennard-Jones potential. The systems were described combining the OPLS-all atom force field^51–53^ (OPLS in the following) and a specific force field (FF-GNP in the following) developed for the description of [Au_25_(SCH_2_CH_2_Ph)_18_]^−1^ by Brancolini *et al.*,^23^ which is fully compatible with OPLS. While for the system AuPh we could directly use FF-GNP, we had to combine the two force fields to obtain an accurate parameterization for systems AuC_2_ and AuC_16_. Indeed, FF-GNP contains precise descriptions of the atomic interactions involving Au, S, and the first C and H atoms of the substituents but does not incorporate any indications about the C and H atoms of the alkane chains farther from the nanocluster kernel. Thus, we decided to join the two force fields as follows:

• AuC_2_: bond interactions were fully parametrized according to FF-GNP, except for the last carbon and relative hydrogen atoms of the ethyl chains which were parametrized with common CT and HC OPLS parameters for carbon and hydrogen atoms respectively. All non-bond interaction parameters come from FF-GNP.

• AuC_16_: bond and non-bond interactions involving gold, sulphur and the first carbon and hydrogen atoms were parametrized according to FF-GNP. Regarding the rest of the alkyl chains, bond interactions come from OPLS using CT as the carbon type and HC as the hydrogen type. Different parametrizations were chosen for the non-bond interactions. In particular, the first two carbons of the chains (and relative hydrogens) were parametrized with FF-GNP while the others have the standard CT and HC OPLS non-bonded interaction parameters.

The solvent (dichloromethane) was parametrized with the standard OPLS force field.^54^ In all calculations the timestep size was set to 1 fs, neighbour list was updated every 20 timesteps and cut-off distances used to calculate non-bonding interactions, were set to 1.20 nm. The simulations were performed at the temperature of 300 K and a pressure of 1 bar by adopting velocity-rescale^55^ and Parrinello–Rahman algorithms respectively.^56^ The two nanoclusters were initially set at a distance of 1.5 nm while starting velocities were randomly generated from a Maxwell–Boltzmann distribution. Hydrogen bonds were constrained with a LINCS algorithm.^57^ Different box cells were used for the three simulations, due to the different dimension of the three systems: in particular 8 × 8 × 8 nm^3^ boxes were used for systems AuPh and AuC_2_, while for the system AuC_16_ we adopted a 15 × 15 × 15 nm^3^ box. These dimensions assured the presence of a 2 nm layer of solvent between the original clusters and their periodic replicas, which is sufficient to prevent artifacts given by replica interactions. Standard metadynamics^39^ calculations were performed on the three systems, using as a collective variable the distance *D*, between the centre of mass of the two Au_13_ cores. Since gold is much heavier than other atoms involved (S, C and H), this value is representative of the distance between the centres of mass of the two MPCs, with the benefit of being not affected by the reorientation of the extended ligands. The potential bias was modelled as Gaussian functions which were deposited every 1 ps, starting from a height of 0.5 kJ mol^−1^ and a width of 0.05 nm that were decreased to 0.1 kJ mol^−1^ and 0.01 nm after 100 ns in order to smoothly refine the shapes of the profiles. Convergence on the free energy profiles was achieved at different simulation lengths, due to the different complexities of the three systems. In particular, we ran 250 ns, 180 ns and 280 ns long simulations for systems AuPh, AuC_2_ and AuC_16_ respectively. Each profile was obtained as the average over a sample of 50 representative profiles, obtained during the last 50 ns of the trajectories. More details about this procedure are available in the ESI.† Finally, in order to explore the effect of the force field on the free energy landscape, we ran a 200 ns long metadynamics calculations of the system AuC_2_ using the AMBER-based force field developed for these MPCs.^22^ The results are reported in the ESI.†

## Conflicts of interest

There are no conflicts to declare.

## Supplementary Material

NA-002-D0NA00213E-s001
