# Supplementary material for: Atomistic insight into the aggregation of [Au25(SR)18]q nanoclusters
Source: Nanoscale Adv. 2020 May 18;2(7):2842–52. doi: 10.1039/d0na00213e (PMC9417423; doi:10.1039/d0na00213e)
Supplement: NA-002-D0NA00213E-s001 [file NA-002-D0NA00213E-s001.pdf]

Electronic Supplementary Material (ESI) for Nanoscale Advances.
This journal is © The Royal Society of Chemistry 2020

Electronic Supplementary Information

Atomistic insight on the aggregation of $[Au_{25}(SR)_{18}]^q$ nanoclusters

*Mirko Vanzan[a], Marta Rosa[a] and Stefano Corni[a, b, *]*

[a]Department of Chemical Sciences, University of Padova, Via Marzolo 1, 35131 Padova, Italy

[b]CNR Institute of Nanoscience, Center S3, via G. Campi 213/A, 41125, Modena, Italy

Insight on π-π stacking interaction in system *AuPh*

As discussed in the main text, system *AuPh* present a shallow minimum at D = 1.20 nm. This minimum corresponds to a dimeric phase where the phenyl rings of the two nanostructures, interact via π-π stacking. As visible form Figure S1, in this configuration the distance between faced phenyl rings is about 0.42 nm, a value even smaller than the distance among faced benzene rings in the gas phase.[1]

Minimum *1-AuPh*

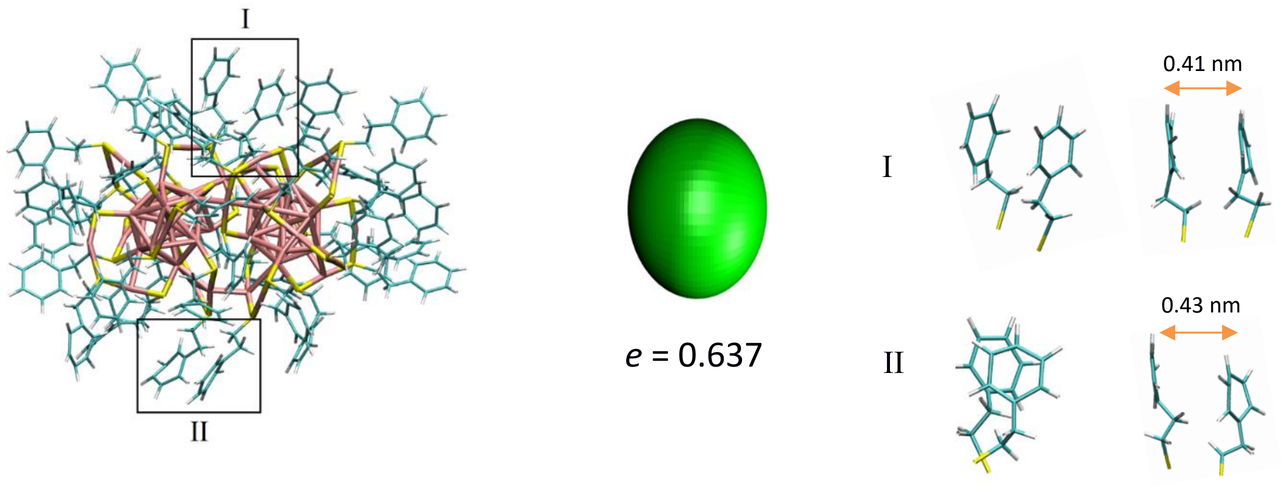

Figure S1. Snapshot of system *AuPh* in its minimum 1-*AuPh* (D = 1.20 nm). Solvent molecules are omitted for clarity. The picture contains an ellipsoid model of the nanoclusters, which eccentricity *e* was calculated as the average of the instantaneous clusters' eccentricities.

Potential energy surface of system AuPh in vacuo

In order to explore the relative contribution the attractive π-π stacking interactions have on the fes of system AuPh, compared to the repulsive coulombic interaction, we calculated through an MD simulation, the potential energy surface of a system composed on two $[Au_{25}(SCH_2CH_2Ph)_{18}]^{-1}$ nanoclusters in vacuo. From this simulation we extracted the total energy trend with respect to the relative nanoclusters distance. The result of this analysis is reported in Figure S2. Here it is evident how the coulombic repulsion dominates over the π-π stacking interactions, as confirmed by the high accuracy of the hyperbolic fit, which indicates a typical coulombic tendency of the potential energy.

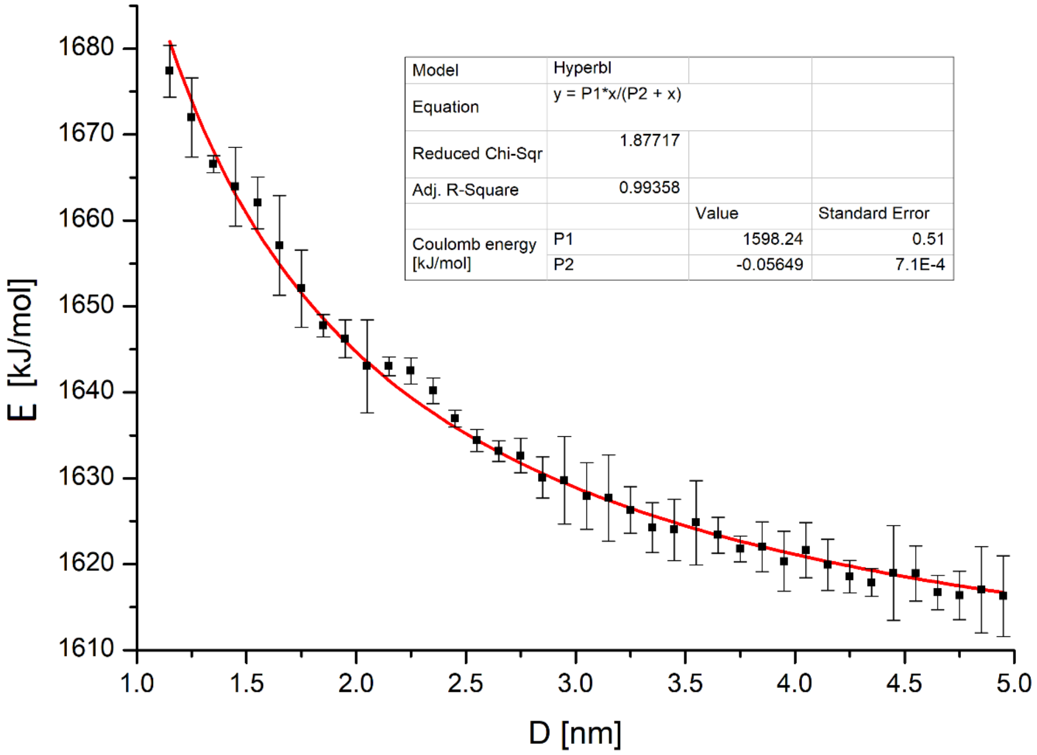

Figure S2. Potential energy surface with respect to the mutual cluster distance for a system composed on two $[Au_{25}(SCH_2CH_2Ph)_{18}]^{-1}$ nanoclusters in vacuo. Hyperbolic fit is pictured in red line.

SASA analysis

To quantify the loss of nanocluster-solvent contact area when the two nanoclusters approach and generate a dimeric structure, we here report the value of the nanocluster Solvent Accessible Surface Area (SASA) with respect to the mutual cluster interdistance for system AuPh. As visible from Figure S3, there is a net loss of SASA when the nanoclusters are interacting and this reflects into a loss of stabilization given by the nanocluster's solvation, which is maximum when the two clusters are isolated.

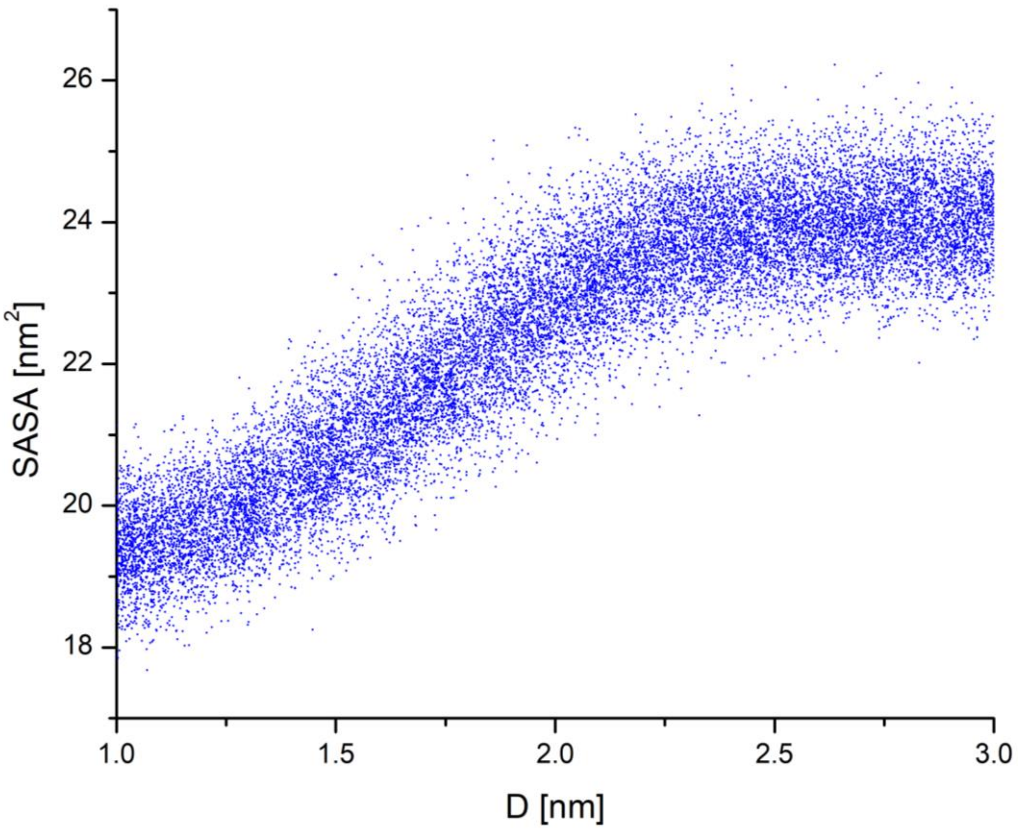

Figure S3. Solvent Accessible Surface Area (SASA) of one of the two $[Au_{25}(SCH_2CH_2Ph)_{18}]^{-1}$ nanocluster of AuPh nanoclusters system with respect to the mutual cluster-cluster distance

Results obtained with AMBER-compatible Force Field

In order to test the reliability of our calculations, we performed a metadynamics simulation on *AuC_2* system using a different force field. Among all the literature available force fields developed for MPCs, we decided to employ the ones proposed by Pohjolainen et. al. in 2016 which is based on AMBER.[2] During these calculations we adopted the same conditions employed for the other metadynamics simulations, which are described in the computational details section.

At the beginning, the results obtained with this force field were not satisfying at all. Indeed, a visual inspection revealed that after a few nanoseconds of simulations, the force field were not able to maintain the bare structure of the nanoclusters, since cores and staples started to break in smaller pieces and to randomly move inside the simulations box. This because, in the original force field, all gold atoms have the same non-bonding parameters and no explicit bonding interactions among core atoms is specified. Thus, the core should be kept intact only through non-bonding forces; however our simulations demonstrated that the magnitude of this interaction are not high enough to maintain the whole structure integer.

Thus, we modified the force field by splitting the gold atoms of a nanocluster into two groups: the core atoms (which contains 13 of the 25 gold atoms) and the staples atoms (which contains the other 12 gold atoms). Then we manually modified the non-bonding interaction parameters for the 13 gold core atoms by multiplying its strength by 10. With this modification, the structures were kept intact all along the trajectory. The simulation ran for 200 ns and the obtained fes are reported in Figure S4, together with the fes obtained with our OPLS based force field.

As visible from the plot, the two profiles are very different. In particular, the profile obtained with the AMBER-based force field (red line) has its global minimum when the distance between the two nanoclusters is about 1.45 nm, indicating a particularly stable dimeric phase, which is in strong contrast with respect to the profile obtained with OPLS force field (black line). The free energy of this minimum is -9.3 kJ/mol with respect to the non-interacting clusters configuration (D = 3 nm).

We believe that the presence of this deep minimum is a calculation artefact that can be explained considering the following aspects:

1) The 10-fold increasing of the non-bonding interaction among the cores atoms naturally stabilize configurations in which the cores are nearby, as in a short-distance dimeric structure.
2) The original non-bonding parameters of gold atoms (which were adopted to describe the Au atoms of the staples) were obtained in order to reproduce the Au-Au interatomic distance of the bulk, which is much shorter than the average Au-Au distance in an MPC.[3] This obviously leads to a shorter dimer equilibrium distance.

For these reasons we believe that the result obtained with our OPLS-compatible force field is more reliable than the one obtained with the AMBER-based force field.

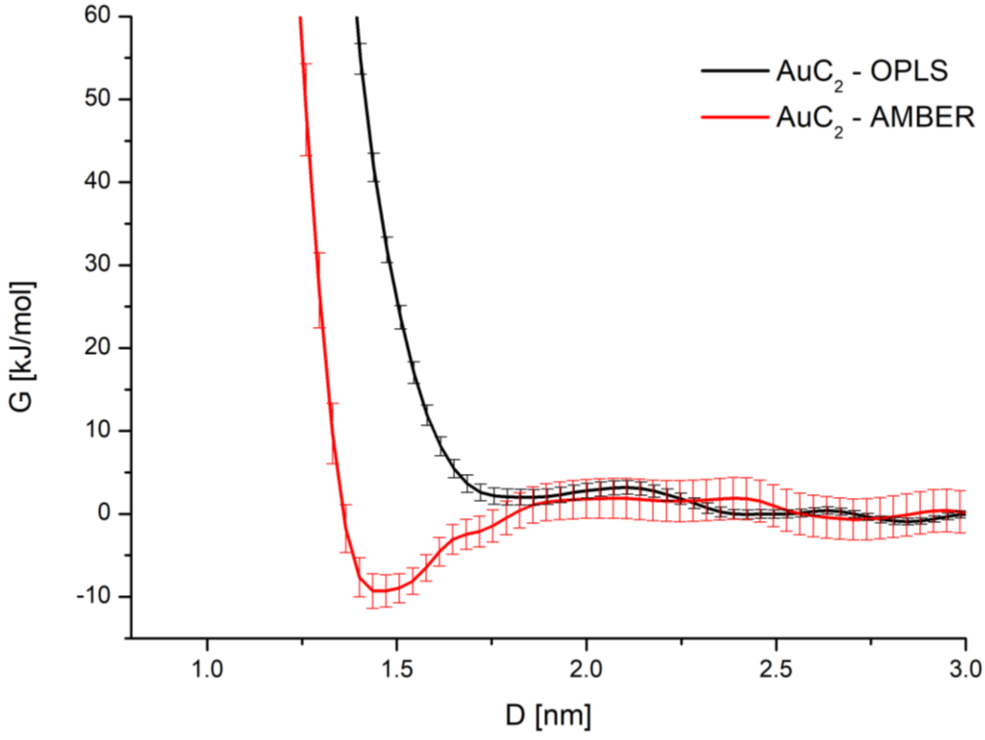

Figure S4. Free energy landscapes of system *AuC_2*, obtained using two different force fields.

Description of the procedure adopted to draw the free energy profiles

In order to assure the convergence of the profiles, we adopted the following checking method. First, for each system we printed 50 fes, relative to the last 50 ns of the trajectory (one for each nanoseconds) and aligned them with the method presented by Tiwary et. al.[4] These allowed us to remove the downshifts on the free energy given by the presence of different amount of hills, while preserving their contribution to the global profiles shapes. The final curves were obtained by averaging these aligned profiles, while the associated error bars represent the standard deviations of the free energy values. In order to check if the obtained profiles were truly representative of the free energy of the systems, we compared them with the ones obtained by averaging the profiles over the last 20, 30 and 40 ns. If all these curves relay within the error bars, the simulations have reached the convergence from 50 ns at least, and the only differences reside on the statistics fluctuations connected to the nanocluster's diffusion. An example of this comparison is given in Figure S5.

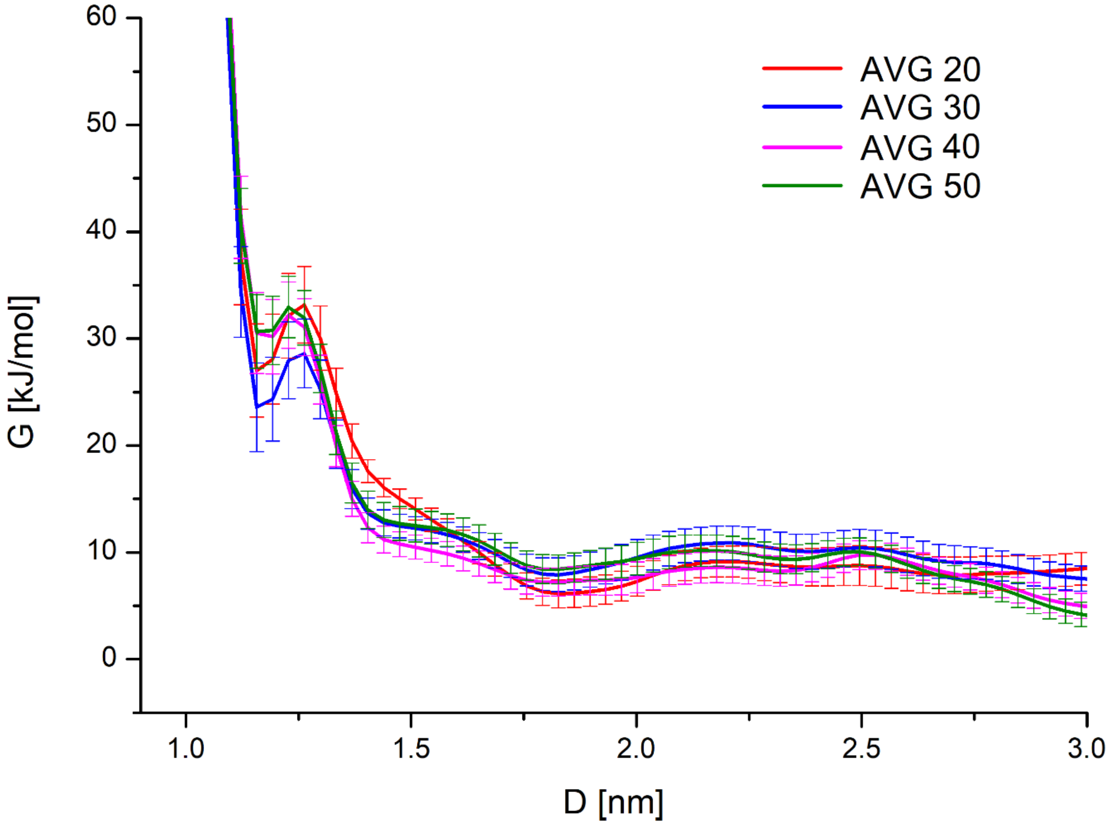

Figure S5. Free energy landscapes of system A, calculated by averaging (AVG) the aligned profiles obtained during the last 20, 30, 40 and 50 ns. Since all curves are included within the error bars, the simulation has reached the convergence.

Bibliography

1 M. O. Sinnokrot, E. F. Valeev and C. D. Sherrill, *J. Am. Chem. Soc.*, 2002, **124**, 10887–10893.

2 E. Pohjolainen, X. Chen, S. Malola, G. Groenhof and H. Häkkinen, *J. Chem. Theory Comput.*, 2016, **12**, 1342–1350.

3 C. Zeng, *Pure Appl. Chem.*, 2018, **90**, 1409–1427.

4 P. Tiwary and M. Parrinello, *J. Phys. Chem. B*, 2015, **119**, 736–742.
